# Characteristics of predicated pathogenic missense variants in human genes for Mendelian disorders

**DOI:** 10.3389/fbinf.2026.1947929

**Published:** 2026-09-30

**Authors:** Ruocen Song, Xiaofei Bai, Xiao Fan

**Affiliations:** 1 Department of Biomedical Engineering, University of Florida, Gainesville, FL, United States; 2 Department of Biology, University of Florida, Gainesville, FL, United States

**Keywords:** genetic and protein properties, linear regression, Mendelian disorders, missense variants, variant pathogenicity

## Abstract

**Background:**

Missense variants represent a major class of genetic variation and contribute substantially to the cause of Mendelian disorders. However, the genetic and protein properties governing their pathogenicity remain incompletely characterized. Existing variant pathogenicity databases, such as ClinVar, are enriched for variants in well-studied genes and therefore may not provide a representative view of pathogenic missense variants across the genome. Meanwhile, recent advances in computational pathogenicity prediction have significantly improved their predictive accuracy, enabling large-scale genome-wide analysis. In this study, we investigated the genetic and protein properties associated with the predicted pathogenicity of missense variants in Mendelian disease genes.

**Methods:**

We collected all possible missense variants in Mendelian genes curated from the Online Mendelian Inheritance in Man (OMIM). Each variant was annotated with pathogenicity predictions from gMVP and a range of properties, including evolutionary conservation, physicochemical substitution characteristics, and functional and structural genetic and protein features. Using multiple linear regression models, we investigated the relationships between the various properties and the predicted pathogenicity of the missense variants. Interaction effects among those properties were also examined. We first validated the quality of gMVP by comparing the effects of each property in the linear regression, fitting to either gMVP pathogenicity predictions or ClinVar pathogenicity labels, on the ClinVar dataset. Then, we extended the analyses to the entire missense dataset to fit gMVP pathogenicity scores. In addition, gene set and robustness analyses were further performed to validate the study findings.

**Results:**

In 655,859 missense variants from ClinVar, 7.1% were labeled as pathogenic/likely pathogenic compared with 6.4% predicted as pathogenic using a gMVP threshold of 0.9. Regression analyses revealed a high degree of concordance between ClinVar and gMVP pathogenicity annotations, with most predictors showing associations in the same direction and of similar magnitude. The genome-wide dataset includes 23,339,549 missense variants in 4,709 Mendelian disease genes, with 31.8% predicted to be pathogenic using a default gMVP threshold of 0.75. Evolutionary conservation, solvent accessibility, and intrinsic disorder were strongly associated with gMVP pathogenicity scores. Other features, including functional annotations and amino acid biochemical properties, also contributed to the predicted pathogenicity with moderate effect sizes. Feature interactions support an integrative model in which conservation and local protein structure jointly influence the variant impact. GO-stratified and other gene-set analyses indicated that these associations were broadly conserved across gene functional contexts and inheritance patterns.

**Conclusion:**

gMVP-predicted pathogenic missense variants are consistently enriched in conserved and structured protein core regions, regardless of gene function and inheritance pattern. These findings highlight the importance of incorporating genetic and protein annotations into variant interpretation frameworks.

## 1 Introduction

Missense variants, which result in single amino acid substitutions, represent one of the most common classes of coding genetic variation in the human genome (Chong et al., 2015; Cooper and Shendure, 2011). While most missense variants are benign, a small subset disrupts protein function and leads to Mendelian diseases, making the accurate identification of the pathogenic variants a central challenge in human genetics and precision medicine (Richards et al., 2015). Despite substantial advances in variant pathogenicity prediction, interpreting the factors underlying these predictions remains a major challenge (Starita et al., 2017; Grimm et al., 2015).

Current pathogenicity prediction methods leverage diverse sources of information, including evolutionary conservation, population allele frequencies (AF), protein sequence and structural information, and predictions from other computational methods (Ng and Henikoff, 2001; Adzhubei et al., 2010; Kircher et al., 2014; Sundaram et al., 2018). Recent methods, including EVE (Frazer et al., 2021), AlphaMissense (Cheng et al., 2023), Evo 2 (Brixi et al., 2026), and protein language model-based predictors (Brandes et al., 2023), have demonstrated improved predictive performance across diverse datasets. However, they often operate as black boxes, producing accurate predictions without providing clear biological interpretation. As a result, the extent to which specific protein properties drive pathogenicity predictions remains unclear. This gap limits our understanding of how missense variants exert their effects and constrains the interpretability of computational models.

While prior studies have demonstrated that pathogenic missense variants tend to be enriched in conserved or structurally constrained regions (Samocha et al., 2017; MacGowan et al., 2024), these analyses have typically focused on individual features or limited feature sets. Protein domains, binding interfaces, regulatory motifs, post-translational modification (PTMs), and 3D structures are all critical for protein stability, molecular interactions, and biological activities (Chothia, 1992; Pawson and Nash, 2003; Neduva and Russell, 2005; Walsh et al., 2005). Variants affecting these critical regions are more likely to disrupt protein function than variants occurring in structurally flexible or functionally less important regions (Yue et al., 2006). In addition, amino acid physicochemical properties, such as hydropathy, charge, molecular weight, and solvent accessibility, may further modulate the functional impact of missense substitutions (Dill, 1990; Rost and Sander, 1994; Tokuriki and Tawfik, 2009; Stein et al., 2019). Systematic, genome-scale quantification of how multiple genetic and protein structural and functional features jointly shape missense pathogenicity across Mendelian disease genes remains limited. Moreover, interaction effects between these properties have rarely been explicitly modeled, and the consistency of these protein determinants across different functional contexts has not been comprehensively evaluated.

Here, we address these gaps by performing a comprehensive, genome-wide analysis of missense variants across Mendelian disease genes. We compiled all possible missense variants in the human reference genome and annotated them with pathogenicity predictions, genetic, and protein annotations. By jointly modeling these features, explicitly testing their interaction effects, and evaluating consistency across gene subgroups and random subsets of variants, we provide a systematic framework for understanding the genetic and protein determinants of missense variant pathogenicity.

## 2 Materials and methods

The analysis pipeline consisted of variant collection, pathogenicity annotation, genetic and protein feature mapping, and statistical modeling (Figure 1).

**FIGURE 1:**
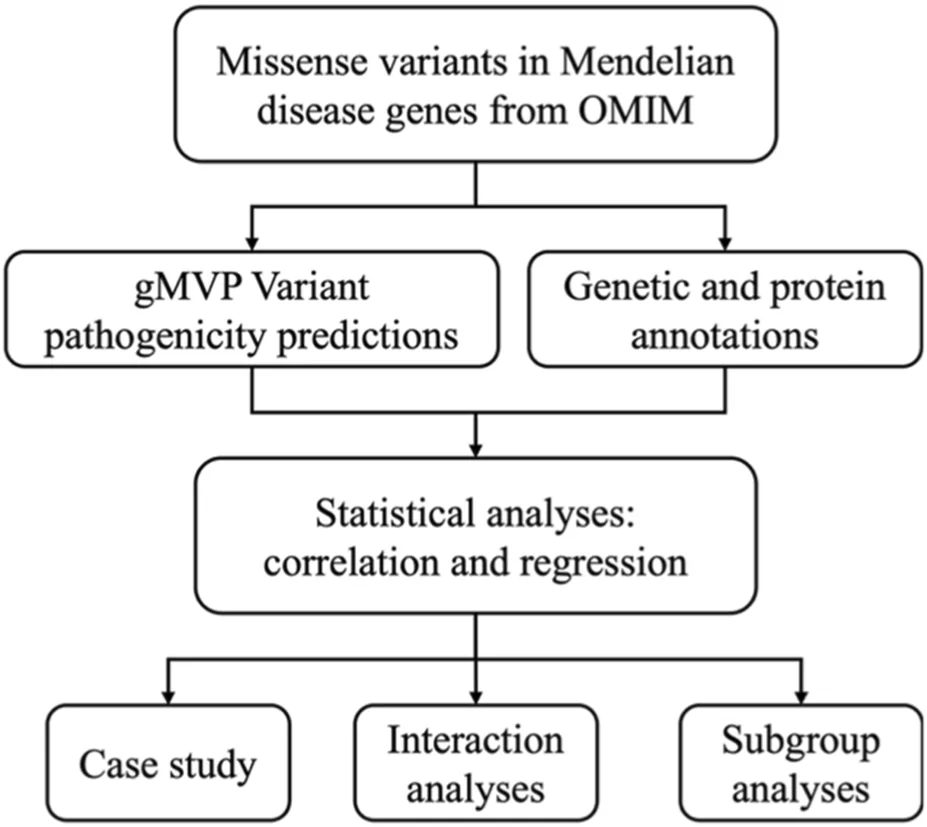
Overview of the study design and analysis workflow.

### 2.1 Datasets

Mendelian disease genes were curated from the Online Mendelian Inheritance in Man (OMIM) database (Amberger et al., 2015). We annotated the pathogenicity of all possible missense variants in these genes using the Graphical Missense Variant Pathogenicity Predictor (gMVP) (Zhang et al., 2022). gMVP was developed using graph attention neural networks to capture both evolutionary and co-evolutionary features and is among the best-performing predictors (Livesey and Marsh, 2022). We used its rank scores for correlation and regression analyses, and its binary classifications to estimate the percentage of predicted pathogenic variants and to compare it with ClinVar pathogenicity labels. All variants across the entire genome were ranked and normalized to [0, 1], with one indicating the most likely pathogenicity. We used the default gMVP rank score greater than 0.75 to estimate the percentage of pathogenic variants. A set of amino acid-level annotations was also collected for each missense variant, based on the canonical transcript in UniProt (The Ensembl Variant Effect Predictor).

Physicochemical properties of both reference and alternate amino acids were annotated based on the amino acid physical property table from the Thermo Fisher Scientific Pierce Protein Methods resource (Kyte and Doolittle, 1982), including hydropathy (Kyte and Doolittle, 1982), charge (Grantham, 1974), and molecular weight (Zimmerman et al., 1968). Hydropathy was categorized as hydrophilic, moderately hydrophobic, or hydrophobic, and charge as positive, neutral, or negative. Hydropathy, charge, and weight changes were calculated as the difference between the alternate and reference amino acids and were used in the downstream analyses. Amino acid substitution similarity was measured using the BLOSUM62 substitution score (Henikoff and Henikoff, 1992).

Evolutionary conservation was assessed using Eigen scores (Davydov et al., 2010; Ionita-Laza et al., 2016). Population AFs were obtained from gnomAD3 (maximum population AF) and the UK Biobank (Chen et al., 2024; Bycroft et al., 2018). Missing population AFs were imputed as 10^–6^. Negative log-scaled AFs were calculated to represent selective constraint and population-level rarity in the analyses.

Protein structural features, including secondary structure and solvent accessibility (Rost and Sander, 1994; Dictionary of protein secondary structure, 1990), were predicted by DSSP (Kabsch and Sander, 1983) based on AlphaFold2-predicted protein 3D structures. For secondary structure, the DSSP-produced eight categories were grouped into three: helix (α-helix, 3-helix, and 5-helix), strand (isolated β-bridge and extended β-strand participating in a β-ladder), and other conformations (hydrogen-bonded turn, bend, and unassigned residues). These grouped secondary-structure annotations were then one-hot encoded. During the regression analyses, the group of other conformations was excluded from the model because it was perfectly collinear with the linear combination of the helix and strand groups, resulting in a singular design matrix. Solvent accessibility was normalized into relative solvent accessibility (RSA) using the same procedure as previous studies (Matveev et al., 2023) within each protein using min-max scaling. Missing solvent accessibility values were imputed using the mean value within the protein.

Protein functional and other structural annotations were obtained from UniProt and included protein domains, binding sites, PTMs, sequence motifs, and intrinsically disordered residues (The UniProt Consortium, 2025). All annotations were manually curated, except for intrinsic disorder, which was predicted using MobiDB (Necci et al., 2017), an ensemble method that combines outputs from multiple individual predictors. All variants were annotated with the above features, which were normalized by their absolute maximum value. This unified dataset served as the basis for our main statistical analyses.

### 2.2 Quality check

We validated the quality of gMVP predictions by comparing feature contributions to both clinically curated and gMVP-predicted pathogenicity on the ClinVar dataset, including variants classified as pathogenic/likely pathogenic or benign/likely benign (Landrum et al., 2016). To match the clinical variant classification standard, which requires a precision greater than 0.9 for a likely pathogenic variant, we used a gMVP rank score greater than 0.9 as the cutoff for comparison with ClinVar pathogenicity labels.

### 2.3 Statistical analysis

#### 2.3.1 Correlation analysis

gMVP rank scores were utilized in all downstream analyses. Spearman’s correlation analysis was used to assess monotonic associations among gMVP rank scores and various genetic and protein features (Haigh and Conover, 1981; Mukaka, 2012). This approach also identifies pairwise correlation among the features we collected at the genome scale.

#### 2.3.2 Regression analysis

Regression analysis (Gelman and Hill, 2007) was used to model missense variant pathogenicity using genetic and protein features and to estimate their contributions. In addition, we incorporate interaction terms between all pairs of features to capture potential non-additive effects. To assess gene functional context-specific effects, separate regression analyses, with and without interaction terms, were performed within three GO categories (The Gene Ontology Consortium, 2026): Biological Process (BP), Molecular Function (MF), and Cellular Component (CC). To determine whether the findings generalized based on inheritance patterns, we repeated the gMVP-based analyses in genes with dominant inheritance. The inheritance patterns were obtained from the OMIM database. Given the large number of variants in the genome-wide analysis, we additionally evaluated the robustness of the regression results in subsets of variants. We repeated the regression analyses 10 times using randomly sampled 10,000 variants, summarizing the mean and standard deviation of the regression coefficients and the consistency of statistical significance (*P* value <0.05) across the 10 repeats.

## 3 Results

### 3.1 Dataset

#### 3.1.1 Distribution of gMVP scores

After applying reference version control, transcript harmonization, and variant-type filters, a total of 23,339,549 missense variants across 4,709 Mendelian disease genes in OMIM were collected. The distribution of gMVP rank scores is shown in Figure 2A. The median gMVP rank score was 0.584 (interquartile range, 0.337–0.809), with a mean of 0.566 (standard deviation (SD) = 0.277) and 31.8% as predicted pathogenic variants with gMVP rank scores above 0.75. The proportion of predicted pathogenic variants varied substantially across genes, from nearly 0% to nearly 100%, as shown in Figure 2B. Most genes contained a relatively small proportion of predicted pathogenic variants, whereas only a minority of genes exhibited a high predicted pathogenic variant burden, resulting in a unimodal distribution with a pronounced right tail. gMVP pathogenicity predictions based on a rank score above 0.75 are expected to classify 25% of missense variants as pathogenic. However, many genes exhibited a moderate to high proportion of predicted pathogenic variants, indicating that many Mendelian genes are conserved and that their variants are more likely to be pathogenic. The high percentage of predicted pathogenic variants does not imply a correspondingly high burden of disease-causing variants. In recessive diseases, the disease occurs only when both alleles carry pathogenic variants.

**FIGURE 2:**
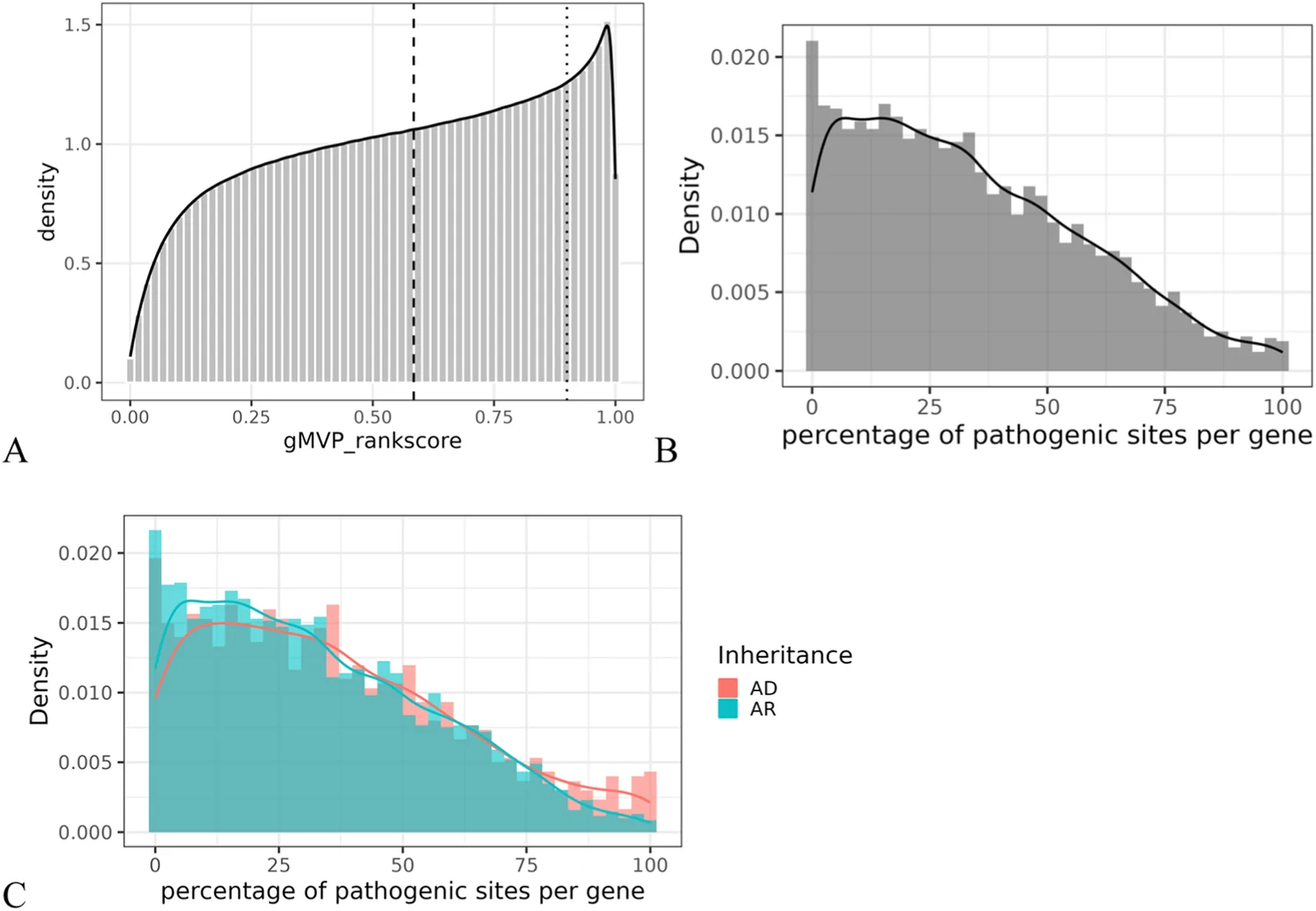
**(A)** Distribution of gMVP rank scores across missense variants in Mendelian disease genes. **(B)** Distribution of the proportion of predicted pathogenic variants per gene across Mendelian disease genes. **(C)** Comparison of predicted pathogenic variant proportion per gene between autosomal dominant (AD) and autosomal recessive (AR) genes.

To determine whether this gene-level heterogeneity of pathogenic variant burden differed by inheritance pattern, we next compared proportions of predicted pathogenic variants between autosomal dominant (AD) and autosomal recessive (AR) genes (Figure 2C). This analysis included 1,172 AD genes and 3,537 AR genes. AD genes showed a slightly higher proportion of predicted pathogenic variants than AR genes, with a mean of 35.8% compared with 32.5%. Despite this moderate shift, the AD and AR distributions were highly similar, indicating that the inheritance pattern alone does not fully explain gene-level differences in predicted pathogenic variant burden. Instead, the broad variability within both inheritance groups suggests that additional genetic and protein properties likely contribute to differences in susceptibility to missense variants across Mendelian disease genes.

#### 3.1.2 Association between pathogenic variant proportion and protein size

We examined the relationship between the proportion of predicted pathogenic variants and protein size. The distribution is shown in Figure 3A. The exponential decay model estimated a negative slope of −0.027 per 100 amino acids. Similarly, we observed a weak negative correlation between the proportion of predicted pathogenic variants and the total number of missense variants per protein (Spearman ρ = −0.126) in Figure 3B. The small effect size indicates that protein length or variant count does not account for the substantial heterogeneity of predicted pathogenic variant burden observed across genes. Therefore, the proportion of predicted pathogenic variants likely reflects intrinsic gene-level properties.

**FIGURE 3:**
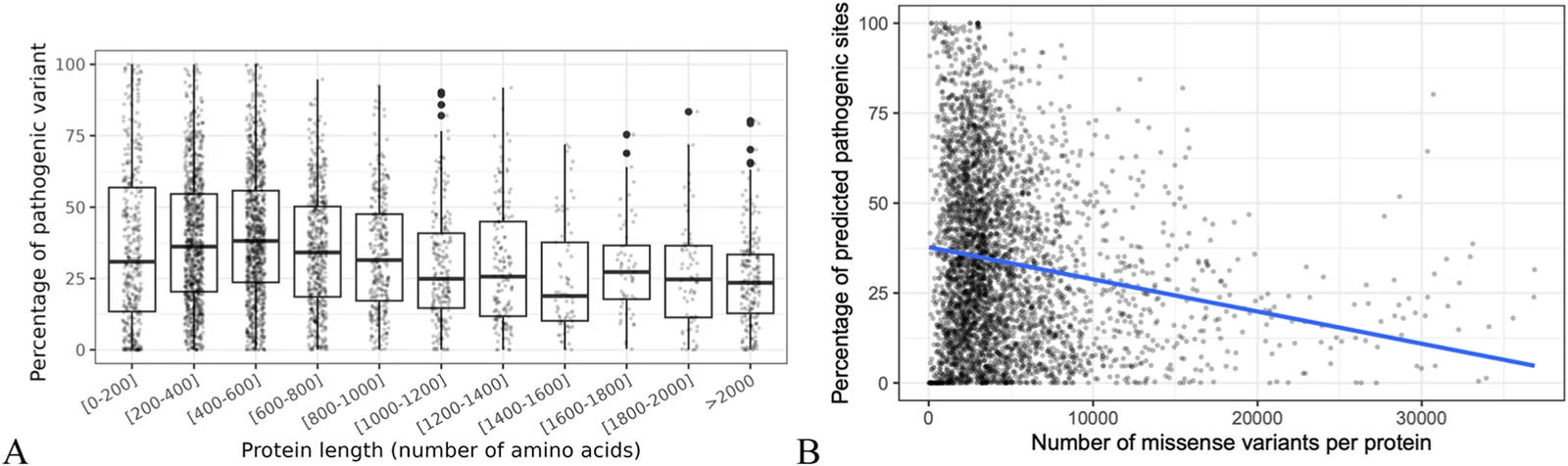
Association between protein length, missense variant count, and the proportion of predicted pathogenic sites. **(A)** Distribution of the percentage of pathogenic variants across protein length, grouped into 200-amino-acid intervals. Boxplots show the median and interquartile range, with individual proteins represented by gray points. **(B)** Relationship between the total number of missense variants per protein and the percentage of predicted pathogenic sites for individual proteins. The blue line represents the fitted linear regression, indicating an overall negative association between missense variant count and predicted pathogenic proportion.

#### 3.1.3 Proportion of pathogenic variants across gene Ontology categories

To evaluate whether predicted pathogenic variant burden differs across broad functional contexts, we compared the portion of predicted pathogenic variants in the three primary GO categories: BP, CC, and MF. The number of proteins and missense variants represented in each GO term-specific subset is summarized in Supplementary Table S1. Figure 4 shows modest variability in the proportion of predicted pathogenic variants across GO categories. These findings suggest that gene-level predicted pathogenic variant burden is not primarily determined by broad GO functional categories, but rather by finer-grained structural and functional features that shape the landscape of missense pathogenicity.

**FIGURE 4:**
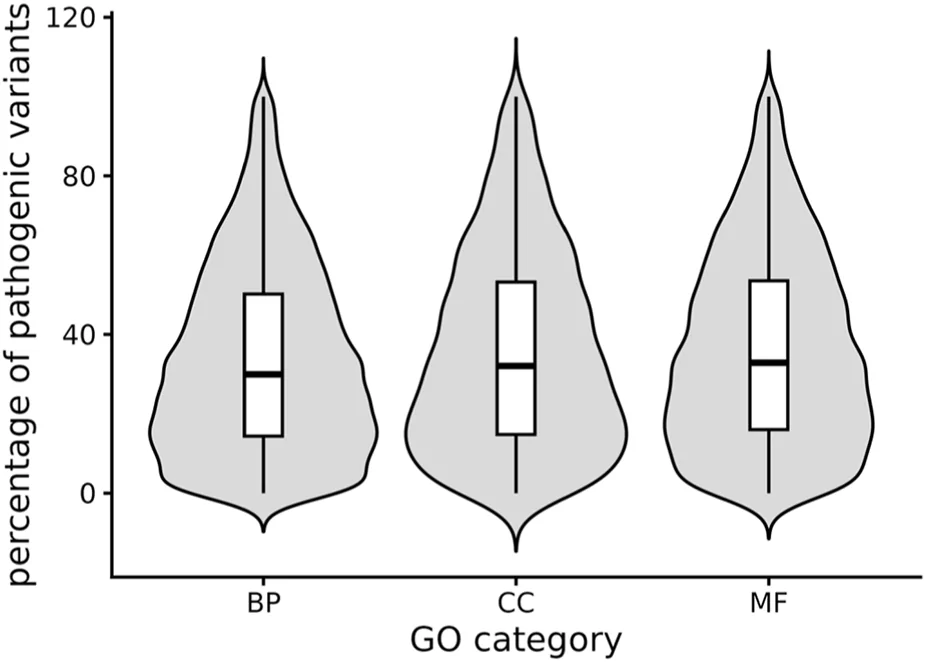
Distribution of predicted pathogenic variant proportion per protein across GO categories.

### 3.2 Consistency between ClinVar and gMVP-predicted pathogenicity

The ClinVar dataset includes 655,859 unique missense variants across 2,862 genes, of which 46,549 (7.1%) are pathogenic/likely pathogenic. gMVP predicts 42,194 variants (6.4%) as pathogenic with a rank score greater than 0.9, comparable to the fraction identified by ClinVar labels.

Before investigating feature associations with gMVP pathogenicity at the genome scale, we first assessed whether gMVP pathogenicity was concordant with clinically curated pathogenicity from ClinVar. Figure 5 shows that the genetic and protein features were consistently correlated with ClinVar and gMVP pathogenicity in the ClinVar dataset. Eigen scores, solvent accessibility, motif, and binding sites were consistently associated with pathogenicity defined by the two methods. Other functional annotations, including BLOSUM scores, secondary structure, AF, and changes in biophysical properties, had little effect on the pathogenicity. These analyses indicate that gMVP provides robust pathogenicity predictions.

**FIGURE 5:**
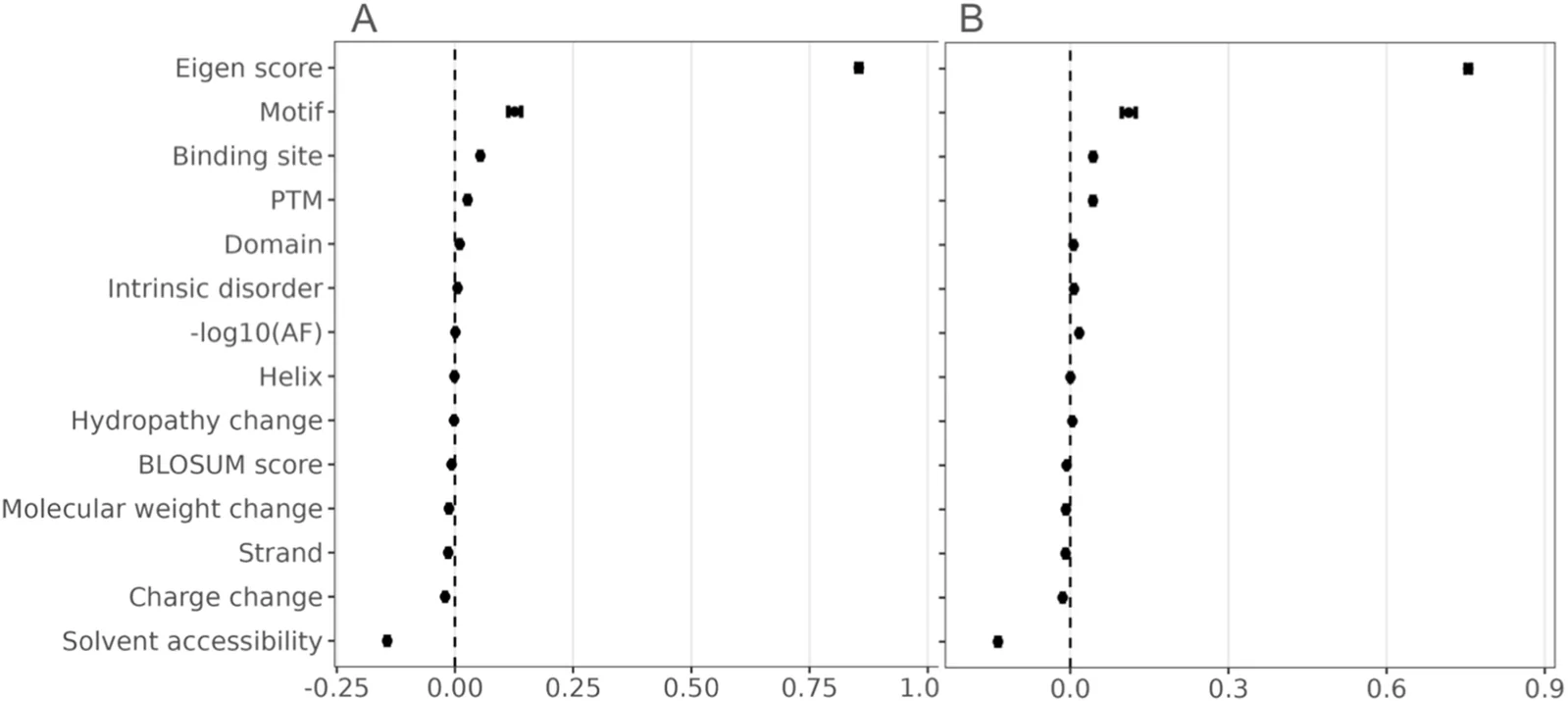
Forest plots of effects of genetic and protein properties on ClinVar and gMVP pathogenicity using linear regression. **(A)** ClinVar binary pathogenicity. **(B)** Binary gMVP pathogenicity using a rank score threshold of 0.9.

### 3.3 Pairwise correlations among gMVP pathogenicity and genetic and protein features

Figure 6A shows the Spearman’s correlation for all pairwise monotonic relationships among the gMVP rank score and various annotated features. Among features correlated with gMVP rank score, Eigen scores showed the strongest positive association (ρ = 0.403), whereas RSA showed the strongest negative association (ρ = −0.396). Intrinsic disorder was also negatively correlated with gMVP rank score (ρ = −0.278). This pattern was not detected in the ClinVar dataset because the number of strictly defined pathogenic variants in intrinsically disordered regions was too small to provide sufficient statistical power. Supplementary Figure S1 shows that variants within and outside intrinsically disordered regions have distinct distributions of gMVP rank scores. Other features correlated with gMVP rank scores include protein domain (ρ = 0.161), strand (ρ = 0.157), and helix (ρ = 0.106).

**FIGURE 6:**
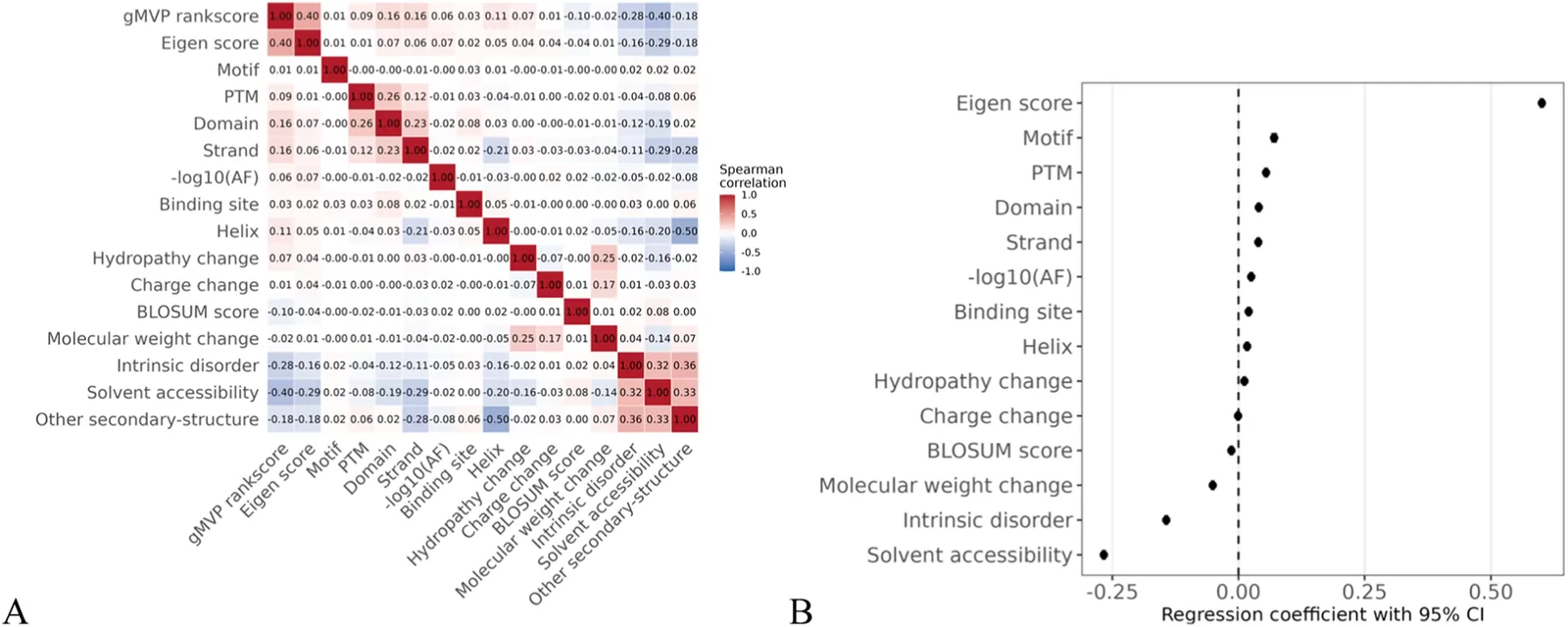
**(A)** Heatmap of the Spearman’s correlation among gMVP rank scores and genetic and protein features. **(B)** Forest plot for effect sizes of the features in the linear regression of gMVP rank scores.

Previous studies have shown that the AlphaFold2 prediction confidence measure, pLDDT, was associated with conformationally flexible regions (Piovesan et al., 2022). Our study also shows a correlation between intrinsic disorder and unstructured secondary structures (ρ = 0.364). Intrinsic disorder was positively correlated with RSA (ρ = 0.319). RSA was negatively correlated with Eigen scores (ρ = −0.289), strand (ρ = −0.293), helix (ρ = −0.196), and domain (ρ = −0.191), indicating that residues buried inside proteins were more often found in constrained, structured, and functional domains.

### 3.4 Associations between genetic and protein features and predicted missense variant pathogenicity using linear regression

To collectively characterize feature associations with the predicted pathogenicity while accounting for other variables, we performed linear regression using all features, including evolutionary conservation, structural, functional, AF, and physicochemical predictors. A forest plot for feature contributions was shown in Figure 6B. After accounting for all other variables, Eigen scores still showed the strongest positive association with the gMVP rank score (β = 0.601), and RSA showed the strongest negative association (β = −0.267), consistent with our correlation analysis. Intrinsic disorder remained negatively associated with predicted pathogenicity (β = −0.143). These three major associations were robust in the subsampling analysis, remaining statistically significant in all 10 independent runs of 10,000 variants (Supplementary Table S2). Several additional features were also associated with gMVP rank scores with smaller effect sizes, including motif (β = 0.071), PTM (β = 0.055), molecular weight change (β = −0.051), and protein domain (β = 0.040). Analyses using gMVP rank scores at the genome level yielded larger effect sizes than those using gMVP binary predictions in the ClinVar dataset, highlighting the power of quantitative measures and a larger dataset. Together, these results indicate that predicted missense pathogenicity is associated with multiple layers of genetic and protein information, including evolutionary conservation, residue exposure, local structure, and functional annotations.

### 3.5 Contributions of feature interactions to missense variant pathogenicity

To systematically evaluate feature associations and interaction effects, we constructed regression models that included interaction terms among all genetic and protein features. Supplementary Table S3 shows that Eigen scores remained the strongest positive predictor (β = 1.051), indicating that conservation is a dominant predictor of pathogenicity even in the presence of feature interaction terms. Several interaction terms showed strong associations with the gMVP rank score, including the interactions between intrinsic disorder and RSA (β = 0.308), which remained statistically significant in all 10 independent subsampling runs of 10,000 variants (Supplementary Table S3). Even though both intrinsic disorder and RSA are negatively correlated with pathogenicity, this positive interaction term suggests that a variant in the structured protein core region does not increase pathogenicity. The same applies to Eigen scores and RSA (β = 0.239); this interaction term counteracts the additive effects of conserved and buried protein regions. Together, these results indicate that predicted missense pathogenicity is shaped not only by strong associations among individual features but also by interactions among structural, functional, evolutionary conservation, and physicochemical features.

### 3.6 Subgroup analyses across gene Ontology categories

To assess whether the determinants of missense pathogenicity depend on gene functional context, we conducted regression analyses stratified by GO categories across MF (Supplementary Table S4), BP (Supplementary Table S5), and CC (Supplementary Table S6). The regression models fitted for each gene subset included the same covariates as the genome-wide interaction model and were used to evaluate whether the major feature associations were preserved across GO functional categories.

Eigen scores remained the strongest and most consistent positive predictor of gMVP rank score, consistent with the genome-wide models. Intrinsic disorder is also a stable determinant of predicted missense pathogenicity regardless of GO categories. In addition, several interaction terms were consistently observed across GO-stratified models. The disorder-solvent accessibility interaction was positive across the MF, BP, and CC subsets. Together, these results indicate that the major determinants of predicted missense pathogenicity are broadly preserved across GO functional contexts.

### 3.7 Targeted subset analyses

Having established that the evolutionary and structural signatures were preserved across GO functional categories, we next examined whether these findings also held true within other gene subsets.

#### 3.7.1 Subgroup analysis in intrinsically disordered genes

Intrinsic disorder and the disorder-solvent accessibility interaction were among the major structural associations identified in the genome-wide regression analyses. We next evaluated whether these effects depended on the inclusion of genes lacking annotated disordered regions. Genes lacking disorder annotations may influence estimates of disorder-associated effects in the genome dataset. To address this possibility, we repeated the analysis in a disorder-restricted dataset comprising 2,189 proteins containing at least one annotated disordered region, thereby excluding proteins that are entirely structured.

Figure 7A shows that intrinsic disorder remained a strong negative predictor (β = −0.123, *P*-value <10^–300^) in the dataset containing only disordered genes. In addition, other predictors show effects similar to those observed in the genome dataset. These results confirm the robustness of our findings. The interaction model also indicated that the disorder-solvent accessibility interaction remained strongly positive (β = 0.316, *P*-value <10^–300^, shown in Supplementary Table S7). The consistency of both the single-variable effects and interaction terms across the genome and disorder-restricted datasets demonstrates that the observed relationships are not driven by completely ordered genes.

**FIGURE 7:**
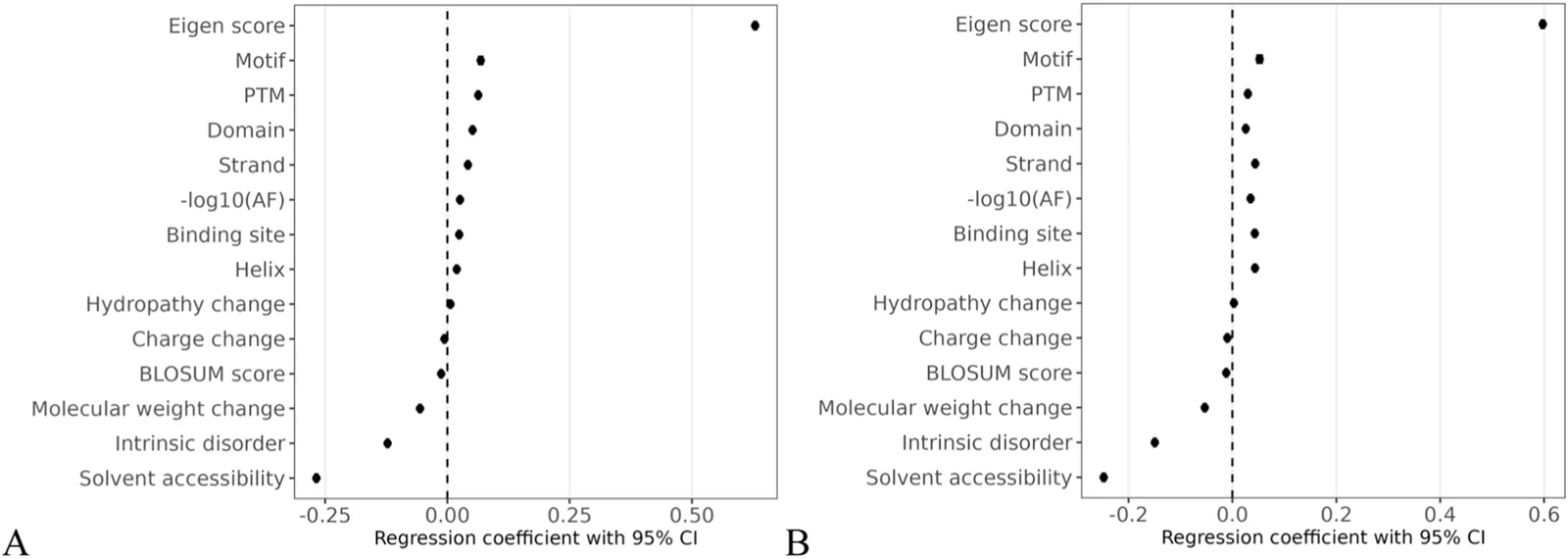
Forest Plot of property effects in the linear regression **(A)** in disordered genes. **(B)** in dominant genes.

#### 3.7.2 Subgroup analyses in dominant genes

To further assess whether the associations observed across the genome dataset were driven by inheritance patterns, we conducted an additional analysis restricted to dominant genes. This subset included 1,173 unique genes and 5,701,973 missense variants. In the dominant-gene regression model (Figure 7B), Eigen scores remained the strongest positively associated feature (β = 0.597, *P*-value <10^–300^). RSA (β = −0.248, *P*-value <10^–300^) and intrinsic disorder (β = −0.149, *P*-value <10^–300^) showed strong negative associations with pathogenicity. The interaction model for the dominant genes further reinforced the relationship between intrinsic disorder and RSA (β = 0.302, *P*-value <10^–300^), shown in Supplementary Table S8. These results indicate that the associations do not depend on the type of gene inheritance.

### 3.8 Case studies

To visualize the relationship between predicted pathogenicity, evolutionary conservation, and protein structural features, we used *KCNJ5* as a representative protein (Figure 8A). This protein encodes a G protein-activated inward-rectifying potassium channel subunit (Choi et al., 2011). In *KCNJ5*, gMVP-predicted pathogenic variants are enriched in the central region of the protein, indicating position-dependent differences in predicted pathogenicity. Evolutionary conservation exhibits a similar pattern, with higher Eigen scores in the central region, suggesting that stronger evolutionary constraint contributes to increased pathogenicity. In contrast, solvent accessibility is negatively correlated with pathogenicity, with the protein center displaying the lowest accessibility scores. Variants located within the intrinsically disordered region consistently have the lowest gMVP rank score across *KCNJ5*. The disordered region also corresponds to the low-confidence segments in the AlphaFold-predicted 3D structure (Figure 8B), located at both termini of the protein. Overall, the *KCNJ5* case study demonstrates that missense pathogenicity is spatially heterogeneous and reflects the combined influence of evolutionary constraint and local protein structural context.

**FIGURE 8:**
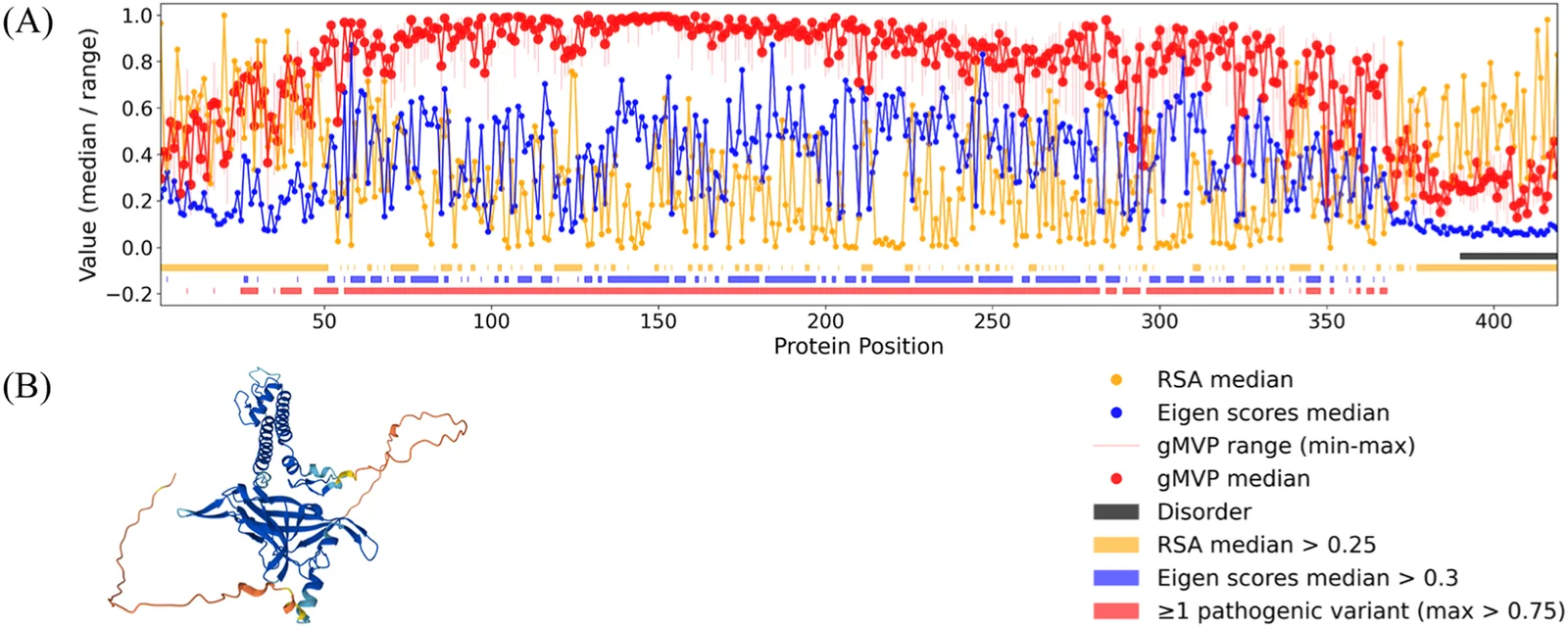
**(A)** Residue-level distribution of gMVP-predicted pathogenicity, Eigen scores, RSA, and intrinsic disorder in *KCNJ5*. **(B)** AlphaFold-predicted *KCNJ5* 3D structure.

## 4 Discussion

In this study, we performed a genome-scale, multi-predictor analysis of missense variant pathogenicity predictions across Mendelian disease genes, leveraging gMVP alongside various structural, functional, evolutionary, and physicochemical annotations. Across more than 23 million missense variants in 4,709 Mendelian disease genes, our results demonstrate that evolutionary conservation and protein structural context are major determinants of missense pathogenicity.

In both pairwise correlation and regression analyses, the gMVP rank score was strongly associated with evolutionary conservation, intrinsic disorder, and solvent accessibility. Variants located in conserved, structured, and protein-core regions were more likely to be pathogenic, whereas substitutions in unconserved, disordered, or protein-surface regions were relatively tolerated. Among other predictors, PTM was also positively associated with pathogenicity, suggesting that functional elements also contribute to pathogenic burden. Importantly, these associations persisted after adjusting for other predictors, including AF, amino acid substitution properties, and functional annotations. These findings indicate that missense pathogenicity is potentially governed by a combination of evolutionary conservation, structural, and functional context.

Subgroup analyses, including the ClinVar dataset, GO-stratified gene sets, dominant genes, and disordered genes, showed a consistent association between gMVP-predicted missense variant pathogenicity and evolutionary conservation, protein structural measures, and their interactions. Overall, these subset analyses support the robustness and biological relevance of these features as determinants of missense variant pathogenicity.

Several limitations of this study should be acknowledged. First, although gMVP demonstrates strong predictive performance, its predictions should not be interpreted as equivalent to clinically established pathogenicity. Second, the evolutionary conservation overlap conceptually with information incorporated into gMVP, but it is the most predictive feature used by almost all variant pathogenicity methods. Third, several protein features, such as solvent accessibility and intrinsic disorder, were derived from computational predictions and are therefore subject to prediction error. Likewise, protein functional annotations from UniProt, such as domains, PTMs, and binding sites, remain incomplete and may contain omissions or inaccuracies. In the future, more accurate AF estimates can be obtained from newer databases with larger sample sizes. Additional analyses incorporating nonlinear modeling may capture higher-order relationships between missense variant pathogenicity and other factors.

## 5 Conclusion

This study provides a quantitative and integrative framework for understanding how gene and protein context shape missense pathogenicity across Mendelian disease genes. By combining correlation, regression, interaction modeling, GO-stratified analyses, gene-subset robustness analyses, and ClinVar-based concordance analyses, we show that missense pathogenicity, as assessed by the gMVP prediction surrogate, is fundamentally shaped by evolutionary conservation and protein structural context. These structural effects are robust across functional categories and consistent between computational predictions and clinically curated datasets. This framework provides a mechanistic basis for interpreting missense variation and for prioritizing potentially disease-causing variants.

## Data Availability

The data associated with this study are available at the following GitHub repository: https://github.com/ruocens/gMVP-missense-variant-analysis.

## References

[B1] Adzhubei I. A. Schmidt S. Peshkin L. Ramensky V. E. Gerasimova A. Bork P. (2010). A method and server for predicting damaging missense mutations. Nat. Methods 7 (4), 248–249. 10.1038/nmeth0410-248 20354512 PMC2855889

[B2] Amberger J. S. Bocchini C. A. Schiettecatte F. Scott A. F. Hamosh A. (2015). OMIM.org: Online Mendelian inheritance in man (OMIM®), an online catalog of human genes and genetic disorders. Nucleic Acids Res. 43 (D1), D789–D798. 10.1093/nar/gku1205 25428349 PMC4383985

[B3] Brandes N. Goldman G. Wang C. H. Ye C. J. Ntranos V. (2023). Genome-wide prediction of disease variant effects with a deep protein language model. Nat. Genet. 55 (9), 1512–1522. 10.1038/s41588-023-01465-0 37563329 PMC10484790

[B4] Brixi G. Durrant M. G. Ku J. Naghipourfar M. Poli M. Sun G. (2026). Genome modelling and design across all domains of life with Evo 2. Nature 652 (8112), 1349–1361. 10.1038/s41586-026-10176-5 41781614 PMC13128491

[B5] Bycroft C. Freeman C. Petkova D. Band G. Elliott L. T. Sharp K. (2018). The UK Biobank resource with deep phenotyping and genomic data. Nature 562 (7726), 203–209. 10.1038/s41586-018-0579-z 30305743 PMC6786975

[B6] Chen S. Francioli L. C. Goodrich J. K. Collins R. L. Kanai M. Wang Q. (2024). A genomic mutational constraint map using variation in 76,156 human genomes. Nature 625 (7993), 92–100. 10.1038/s41586-023-06045-0 38057664 PMC11629659

[B7] Cheng J. Novati G. Pan J. Bycroft C. Žemgulytė A. Applebaum T. (2023). Accurate proteome-wide missense variant effect prediction with AlphaMissense. Science 381 (6664), eadg7492. 10.1126/science.adg7492 37733863

[B8] Choi M. Scholl U. I. Yue P. Björklund P. Zhao B. Nelson-Williams C. (2011). K+ channel mutations in adrenal aldosterone-producing adenomas and hereditary hypertension. Science 331 (6018), 768–772. 10.1126/science.1198785 21311022 PMC3371087

[B9] Chong J. X. Buckingham K. J. Jhangiani S. N. Boehm C. Sobreira N. Smith J. (2015). The genetic basis of Mendelian phenotypes: discoveries, challenges, and opportunities. Am. J. Hum. Genet. 97 (2), 199–215. 10.1016/j.ajhg.2015.06.009 26166479 PMC4573249

[B10] Chothia C. (1992). One thousand families for the molecular biologist. Nature 357 (6379), 543–544. 10.1038/357543a0 1608464

[B11] Cooper G. M. Shendure J. (2011). Needles in stacks of needles: finding disease-causal variants in a wealth of genomic data. Nat. Rev. Genet. 12 (9), 628–640. 10.1038/nrg3046 21850043

[B12] Davydov E. V. Goode D. L. Sirota M. Cooper G. M. Sidow A. Batzoglou S. (2010). Identifying a high fraction of the human genome to be under selective constraint using GERP++. PLOS Comput. Biol. 6 (12), e1001025. 10.1371/journal.pcbi.1001025 21152010 PMC2996323

[B13] Dictionary of protein secondary structure (1990). Pattern Recognition of Hydrogen Bonded and Geometrical Features - Kabsch - 1983 - Biopolymers - Wiley Online Library. 10.1002/bip.360221211 6667333

[B14] Dill K. A. (1990). Dominant forces in protein folding. Biochemistry 29 (31), 7133–7155. 10.1021/bi00483a001 2207096

[B15] Frazer J. Notin P. Dias M. Gomez A. Min J. K. Brock K. (2021). Disease variant prediction with deep generative models of evolutionary data. Nature 599 (7883), 91–95. 10.1038/s41586-021-04043-8 34707284

[B16] Gelman A. Hill J. (2007). Data Analysis Using Regression and Multilevel/Hierarchical Models. Cambridge University Press.

[B17] Grantham R. (1974). Amino acid difference formula to help explain protein evolution. Science 185 (4154), 862–864. 10.1126/science.185.4154.862 4843792

[B18] Grimm D. G. Azencott C. A. Aicheler F. Gieraths U. MacArthur D. G. Samocha K. E. (2015). The evaluation of tools used to predict the impact of missense variants is hindered by two types of circularity. Hum. Mutat. 36 (5), 513–523. 10.1002/humu.22768 25684150 PMC4409520

[B19] Haigh J. Conover W. J. (1981). Practical nonparametric statistics. J. R. Stat. Soc. Ser. Gen. 144 (3), 370–371. 10.2307/2981807

[B20] Henikoff S. Henikoff J. G. (1992). Amino acid substitution matrices from protein blocks. Proc. Natl. Acad. Sci. 89 (22), 10915–10919. 10.1073/pnas.89.22.10915 1438297 PMC50453

[B21] Ionita-Laza I. McCallum K. Xu B. Buxbaum J. D. (2016). A spectral approach integrating functional genomic annotations for coding and noncoding variants. Nat. Genet. 48 (2), 214–220. 10.1038/ng.3477 26727659 PMC4731313

[B22] Kabsch W. Sander C. (1983). Dictionary of protein secondary structure: pattern recognition of hydrogen‐bonded and geometrical features. Biopolym. Orig. Res. Biomol. 22 (12), 2577–2637. 6667333 10.1002/bip.360221211

[B23] Kircher M. Witten D. M. Jain P. O’roak B. J. Cooper G. M. Shendure J. (2014). A general framework for estimating the relative pathogenicity of human genetic variants. Nat. Genet. 46 (3), 310–315. 10.1038/ng.2892 24487276 PMC3992975

[B24] Kyte J. Doolittle R. F. (1982). A simple method for displaying the hydropathic character of a protein. J. Mol. Biol. 157 (1), 105–132. 10.1016/0022-2836(82)90515-0 7108955

[B25] Landrum M. J. Lee J. M. Benson M. Brown G. Chao C. Chitipiralla S. (2016). ClinVar: public archive of interpretations of clinically relevant variants. Nucleic Acids Res. 44 (D1), D862–D868. 10.1093/nar/gkv1222 26582918 PMC4702865

[B26] Livesey B. J. Marsh J. A. (2022). Interpreting protein variant effects with computational predictors and deep mutational scanning. Dis. Model Mech. 15 (6), dmm049510. 10.1242/dmm.049510 35736673 PMC9235876

[B27] MacGowan S. A. Madeira F. Britto-Borges T. Barton G. J. (2024). A unified analysis of evolutionary and population constraint in protein domains highlights structural features and pathogenic sites. Commun. Biol. 7 (1), 447. 10.1038/s42003-024-06117-5 38605212 PMC11009406

[B28] Matveev E. V. Safronov V. V. Ponomarev G. V. Kazanov M. D. (2023). Predicting structural susceptibility of proteins to proteolytic processing. Int. J. Mol. Sci. 24 (13), 10761. 10.3390/ijms241310761 37445939 PMC10342023

[B29] Mukaka M. M. (2012). A guide to appropriate use of Correlation coefficient in medical research. Malawi Med. J. 24 (3), 69–71. 23638278 PMC3576830

[B30] Necci M. Piovesan D. Dosztányi Z. Tosatto S. C. (2017). MobiDB-lite: fast and highly specific consensus prediction of intrinsic disorder in proteins. Bioinformatics 33 (9), 1402–1404. 10.1093/bioinformatics/btx015 28453683

[B31] Neduva V. Russell R. B. (2005). Linear motifs: evolutionary interaction switches. FEBS Lett. 579 (15), 3342–3345. 10.1016/j.febslet.2005.04.005 15943979

[B32] Ng P. C. Henikoff S. (2001). Predicting deleterious amino acid substitutions. Genome Res. 11 (5), 863–874. 10.1101/gr.176601 11337480 PMC311071

[B33] Pawson T. Nash P. (2003). Assembly of cell regulatory systems through protein interaction domains. Science 300 (5618), 445–452. 10.1126/science.1083653 12702867

[B34] Piovesan D. Monzon A. M. Tosatto S. C. (2022). Intrinsic protein disorder and conditional folding in AlphaFoldDB. Protein Sci. 31 (11), e4466. 10.1002/pro.4466 36210722 PMC9601767

[B35] Richards S. Aziz N. Bale S. Bick D. Das S. Gastier-Foster J. (2015). Standards and guidelines for the interpretation of sequence variants: a joint consensus recommendation of the American College of Medical Genetics and genomics and the association for molecular pathology. Genet. Med. 17 (5), 405–423. 10.1038/gim.2015.30 25741868 PMC4544753

[B36] Rost B. Sander C. (1994). Conservation and prediction of solvent accessibility in protein families. Proteins Struct. Funct. Bioinforma. 20 (3), 216–226. 10.1002/prot.340200303 7892171

[B37] Samocha K. E. Kosmicki J. A. Karczewski K. J. O’Donnell-Luria A. H. Pierce-Hoffman E. MacArthur D. G. (2017). Regional missense constraint improves variant deleteriousness prediction. BioRxiv, 148353. 10.1101/148353

[B38] Starita L. M. Ahituv N. Dunham M. J. Kitzman J. O. Roth F. P. Seelig G. (2017). Variant interpretation: functional assays to the rescue. Am. J. Hum. Genet. 101 (3), 315–325. 10.1016/j.ajhg.2017.07.014 28886340 PMC5590843

[B39] Stein A. Fowler D. M. Hartmann-Petersen R. Lindorff-Larsen K. (2019). Biophysical and mechanistic models for disease-causing protein variants. Trends Biochem. Sci. 44 (7), 575–588. 10.1016/j.tibs.2019.01.003 30712981 PMC6579676

[B40] Sundaram L. Gao H. Padigepati S. R. McRae J. F. Li Y. Kosmicki J. A. (2018). Predicting the clinical impact of human mutation with deep neural networks. Nat. Genet. 50 (8), 1161–1170. 10.1038/s41588-018-0167-z 30038395 PMC6237276

[B41] The Ensembl Variant Effect Predictor. Genome Biology | Springer Nature Link. Available online at: https://link.springer.com/article/10.1186/S13059-016-0974-4 (Accessed January 21, 2026). 10.1186/s13059-016-0974-4 PMC4893825 27268795

[B42] The Gene Ontology Consortium (2026). The gene ontology knowledgebase in 2026. Nucleic Acids Res. 54 (D1), D1779–D1792. 10.1093/nar/gkaf1292 41413728 PMC12807639

[B43] The UniProt Consortium (2025). UniProt: the universal protein knowledgebase in 2025. Nucleic Acids Res. 53 (D1), D609–D617. 10.1093/nar/gkae1010 39552041 PMC11701636

[B44] Tokuriki N. Tawfik D. S. (2009). Stability effects of mutations and protein evolvability. Curr. Opin. Struct. Biol. 19 (5), 596–604. 10.1016/j.sbi.2009.08.003 19765975

[B45] Walsh C. T. Garneau‐Tsodikova S. Gatto Jr G. J. (2005). Protein posttranslational modifications: the chemistry of proteome diversifications. Angew. Chem. Int. Ed. 44 (45), 7342–7372. 10.1002/anie.200501023 16267872

[B46] Yue P. Melamud E. Moult J. (2006). SNPs3D: candidate gene and SNP selection for association studies. BMC Bioinforma. 7 (1), 166. 10.1186/1471-2105-7-166 16551372 PMC1435944

[B47] Zhang H. Xu M. S. Fan X. Chung W. K. Shen Y. (2022). Predicting functional effect of missense variants using graph attention neural networks. Nat. Mach. Intell. 4 (11), 1017–1028. 10.1038/s42256-022-00561-w 37484202 PMC10361701

[B48] Zimmerman J. M. Eliezer N. Simha R. (1968). The characterization of amino acid sequences in proteins by statistical methods. J. Theor. Biol. 21 (2), 170–201. 10.1016/0022-5193(68)90069-6 5700434

