# Supplementary material for: Characteristics of predicated pathogenic missense variants in human genes for Mendelian disorders
Source: Front Bioinform. 2026 Sep 30;6:1947929. doi: 10.3389/fbinf.2026.1947929 (PMC13662176; doi:10.3389/fbinf.2026.1947929)
Supplement: Supplementary file 1 [file DataSheet1.pdf]

Characteristics of Predicated Pathogenic Missense Variants in Human Genes for Mendelian Disorders

Ruocen Song, MSc[1], Xiaofei Bai, PhD[2], and Xiao Fan, PhD[1]

[1] Department of Biomedical Engineering, University of Florida, Gainesville, FL, USA.
[2] Department of Biology, University of Florida, Gainesville, FL, USA.

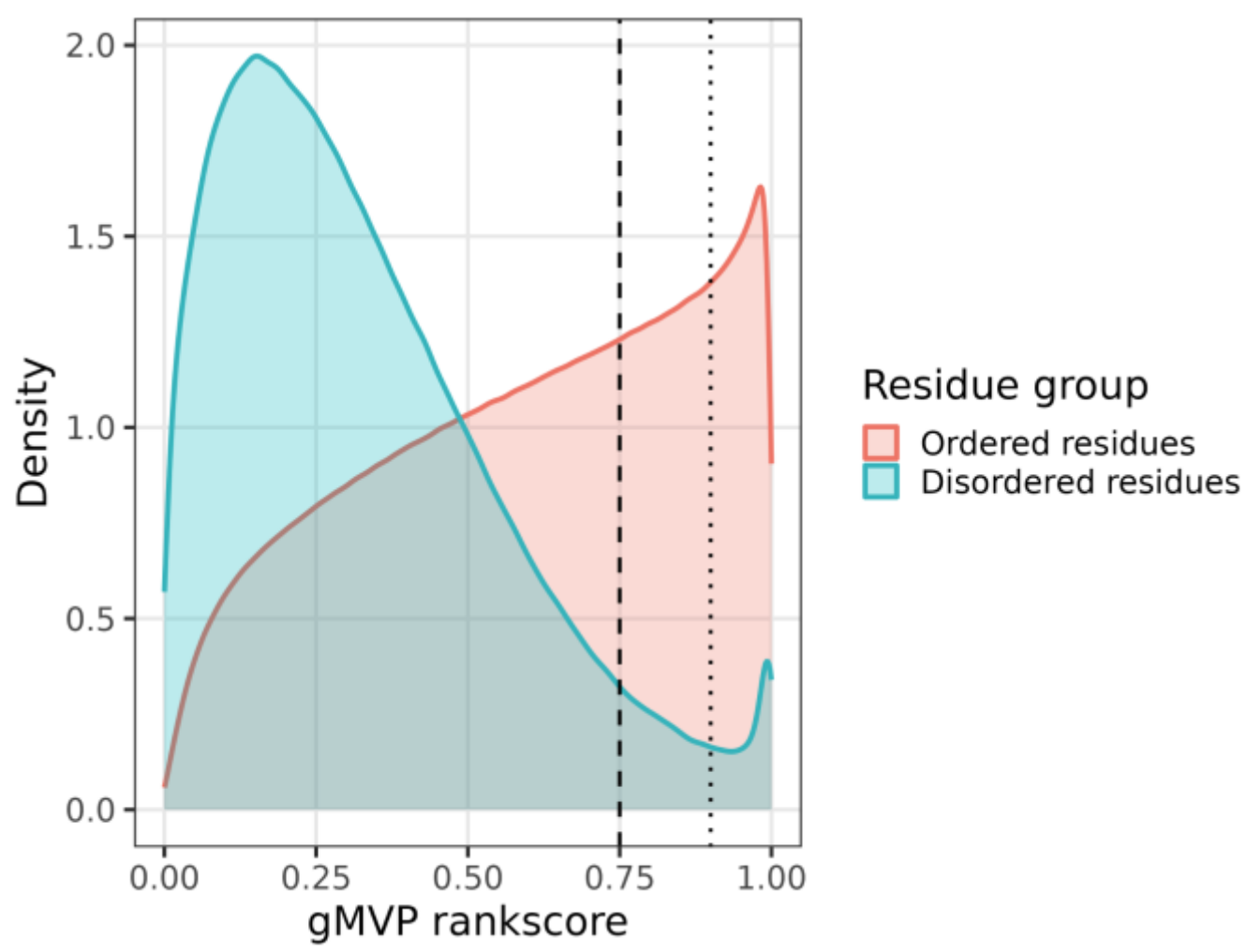

Figure S1. Distribution of gMVP rank scores for missense variants located within ordered and intrinsically disordered regions.
